# Immunopathological features of highly pathogenic Korean Lineage B PRRSV-2: insights into virulence indicators and host immune responses

**DOI:** 10.3389/fimmu.2025.1599468

**Published:** 2025-06-18

**Authors:** Gyeong-Seo Park, Seung-Chai Kim, Hwan-Ju Kim, Chang-Gi Jeong, Sang-Chul Kang, Go-Eun Shin, Seoung-Hee Kim, Hye-Young Jeong, Kyoung-Ki Lee, Sang-Myeong Lee, Won-Il Kim

**Affiliations:** ^1^ Collenge of Veterinary Medicine, Jeonbuk National University, Iksan, Republic of Korea; ^2^ Vaccine Lab, WOOGENE B&G Co., LTD., Seoul, Republic of Korea; ^3^ Biosafety Research Institute, Jeonbuk National University, Iksan, Republic of Korea; ^4^ Animal Clinical Evaluation Center, Optipharm Inc, Cheongju, Republic of Korea; ^5^ Animal Disease Diagnostic Division, Animal and Plant Quarantine Agency, Gimcheon, Republic of Korea; ^6^ College of Veterinary Medicine, Chungbuk National University, Cheongju, Republic of Korea

**Keywords:** porcine reproductive and respiratory syndrome virus, Korean Lineage, pathogenicity, immune characteristics, host immunity, immune checkpoint molecules

## Abstract

**Introduction:**

Porcine reproductive and respiratory syndrome virus (PRRSV) remains one of the most economically devastating pathogens in swine, primarily due to its extensive genetic diversity and lineage-dependent pathogenicity. Despite widespread vaccination, distinct PRRSV-2 lineages continue to circulate in Korea. This study aimed to elucidate the immunopathological features of two Korean-specific Lineage B (LKB) strains, GGYC45 and PJ10, compared with a vaccine-like L5 strain, M8.

**Methods:**

Thirty, 4-week-old piglets were divided into M8-, GGYC45-, PJ10-infected groups, and control. After acclimatization, pigs were intramuscularly inoculated with PRRSV-2 strains. Pigs were monitored, and blood and nasal swabs were collected. At 12- and 28 days post-infection (dpi), pigs were euthanized for histopathological analysis and tissue collection. Histopathological evaluations were conducted on lung and brain tissues. Bronchoalveolar lavage (BAL) cells and lung tissues were analyzed for immune responses, including flow cytometry (FACS), cytokine expression, viral load, and expression of immune checkpoint molecules.

**Results:**

Both LKB strains (GGYC45 and PJ10) observed moderate to severe clinical symptoms. Notably, PJ10-infected pigs exhibited high mortality accompanied by significantly (*p < 0.05*) low average daily weight gain (ADWG), high temperatures, and high levels of viremia and viral loads in various tissues. Immunopathological analysis showed severe respiratory and neurological lesions in PJ10-infected pigs. PJ10 destroyed over 90% of residential alveolar macrophages and increased infiltrated monocyte-derived cells and T lymphocytes in the lungs up to 12 dpi. Pigs infected with the GGYC45 strain exhibited a relatively lower virulence profile than those infected with the PJ10 strain; however, GGYC45 induced moderate pathogenicity in pigs. Regardless of the lineages or genotypes, pigs infected with PRRSV-2 increased immune checkpoint molecule expression, such as PD1, PDL1, CTLA4, IDO1, and LAG3 in BAL cells, resulting in insufficient T cell activation.

**Conclusion:**

These results highlight the differential virulence and immunomodulatory profiles of genetically distinct PRRSV-2 strains circulating in Korea. The heightened immune checkpoint expression, particularly in PJ10-infected pigs, underscores a potential mechanism of PRRSV-induced immune suppression and viral persistence. This study provides critical insights into PRRSV pathogenesis and host-virus interactions and supports the need for lineage-adapted control strategies that account for both the genetic heterogeneity of PRRSV and immune evasion mechanisms.

## 1 Introduction

The porcine reproductive and respiratory syndrome (PRRS) virus has emerged as a formidable and lethal threat to the global swine industry since its initial outbreaks in the United States and Europe (1). The economic repercussions of this disease in the U.S. swine industry alone have been estimated to incur an annual loss of approximately $664 million (2). The causative agent, PRRSV, is a positive-sense single-stranded RNA virus (~15 kb) classified as Betaarterivirus by the International Committee on Taxonomy of Viruses (ICTV, 2018), belonging to the family *Arteriviridae* of the order Nidovirales (3–5). The PRRSV genome comprises at least 10 open reading frames (ORFs), encompassing seven structural genes (ORF1a/b, ORF2a/b, ORF3, ORF4, ORF5/5a, ORF6, and ORF7) and additional nonstructural genes (6). PRRSV is categorized into two divergent genotypes, Betaarterivirus suid-1 (formerly PRRSV-1, EU-type, prototype strain Lelystad virus) and Betaarterivirus suid-2 (formerly PRRSV-2, NA-type, prototype strain VR2332 virus), exhibiting approximately 60% similarity at the nucleotide level (7, 8). According to the ORF5 lineage classification system, PRRSV-2 is subdivided into nine lineages, lineage 1 (L1) to lineage 9 (L9) (9–13). Although the origins of these nation-specific lineages remain largely unknown, Korean lineages have been observed to form a cohesive cluster in whole-genome-based phylograms, exhibiting an identical deletion pattern in the hypervariable region of nonstructural protein 2 (nsp2), suggesting regional independent evolution (14).

Generally, PRRSV-2 strains of different lineages induce varying levels of pathogenicity and susceptibility in infected pigs (15–17). Notably, the past decade has seen an increase in sudden and severe outbreaks caused by highly pathogenic PRRSV-2 strains, such as the emergence of Highly Pathogenic PRRSV (HP-PRRSV) in China and NADC30-like or NADC34-like PRRSV strains (16, 18–22). Since 2014, the emergence of LKB (Korean Lineage B) in Korea is suspected to result from potential recombination between LKC (Korean Lineage C) and MLV vaccine strains, which have been extensively used nationwide for decades (14). Previous studies have conducted pathogenicity experiments on field isolates from Korea, revealing that strains belonging to the LKB (Korean Lineage B) exhibit relatively higher pathogenicity (23).

The immunopathological characteristics of PRRSV are strain-dependent. Upon PRRSV exposure, the virus replicates in alveolar macrophages (AMs) and swiftly disseminates throughout the host. Viremia peaks at approximately 7–10 days post-infection (dpi) and typically subsides by 28 dpi, contingent upon the strains, pig age, and host immune status (24). During high-pathogenic PRRSV infection, the lung environment of pigs undergoes significant alterations and collapse, leading to various local and peripheral immune responses (25–29). Among effector T lymphocytes, cytotoxic T lymphocytes (CTLs), alongside natural killer (NK) cells, play a crucial role in eliminating virus-infected cells. Despite advancements in our understanding of host immunity, the features and role of the immune response, both local and systemic, to genetically unique Korean PRRSV strains remain elusive.

Immune checkpoint molecules serve as negative regulatory receptors expressed on immune cells. Under normal physiological conditions, the host maintains self-tolerance, modulating the breadth of effector immune responses in peripheral tissues (30, 31). However, persistent immune checkpoint signaling leads to T-cell dysfunction, inducing an ‘exhausted state’ in effector immune cells (32, 33). Notable immune checkpoint molecules include programmed cell death 1 (PD1) and its ligand programmed cell death ligand (PDL-1), which are upregulated at various stages of viral infection and contribute to T-cell proliferation (34–36). Other immune checkpoint molecules have also been studied in the context of immunosuppressive viral diseases (37). Recent findings indicate upregulated expression of immune checkpoint molecules during highly virulent PRRSV strain infections, leading to the induction of non-effector T cells (38). However, the role of immune checkpoint molecules in genetically distinct PRRSV strains remains poorly understood.

This study aims to investigate the immunopathological characteristics of genetically unique Korean PRRSV-2 strains, including strain-specific clinical signs and pathology, gross lesions, strain-specific T-cell subset proliferation and differentiation, and expression of immune checkpoint molecules. Thereby, this research contributes crucial insights into highly virulent PRRSV-2 strains prevalent in Korea.

## 2 Materials and methods

### 2.1 Cell culture and virus propagation

The three Korean PRRSV-2 strains, previously categorized into distinct lineages, were obtained from field samples submitted to the JBNU Veterinary Diagnostic Center (JBNU-VDC). The PRRSV-2 (NA-type) strains utilized in this study were JB15-M8-GN (M8, GenBank: AF066183.4), GGYC45 (GenBank: MN073134.1), and JB15-PJ10-GN (PJ10, GenBank: MK057532.1). Strains belonging to Korean Lineage B (LKB), a subgroup of the Korean Lineage that occurs specifically in South Korea, have been consistently reported to account for over 10% of cases annually (39). Phylogenetic tree analyses based on the ORF5 region and whole-genome sequencing (WGS) revealed differing clustering patterns (12, 14). While ORF5-based analysis suggested that strains belonging to LKB grouped within the same clade, WGS analysis demonstrated their separation into distinct clades (**Supplementary Figures 1A, B**). The tested Korean PRRSV-2 strains represent distinct pathogenic phenotypes: M8 is a vaccine-like strain classified under Lineage 5 with low pathogenicity, GGYC45 belongs to Lineage Korean B (LKB) and exhibits moderate virulence, and PJ10, also classified as LKB, has been associated with severe clinical signs, mortality and is considered highly pathogenic based on previous challenge studies (23).

These viruses were propagated in primary porcine alveolar macrophages (PAMs) using RPMI-1640 medium (Gibco^®^ RPMI-1640, Life Technologies, Carlsbad, USA) supplemented with 10% heat-inactivated fetal bovine serum (FBS, Capricorn Scientific), 2 mM L-glutamine, and an antibiotic-antimycotic cocktail (Anti-Anti, Life Technologies) containing 100 IU/ml penicillin, 100 µg/ml streptomycin, and 0.25 µg/ml Fungizone^®^ [amphotericin B] at 37°C in a humidified 5% CO_2_ environment. This culture medium is referred to as RPMI growth medium. Viral titration was conducted on PAM cells, and the titers were determined at 5 to 6 days post-inoculation based on cytopathic effects (CPE), expressed as TCID_50_/mL (40). The virus-containing cell lysate was clarified by centrifugation at 4000 rpm for 30 min at 4°C. Following virus propagation, the cell lysate containing the virus was clarified via centrifugation at 4000 rpm for 30 minutes at 4°C. The clarified samples were filtered through a 0.22μm-pore size filter and collected in a sterile container.

### 2.2 Animal study

**Figure 1:**
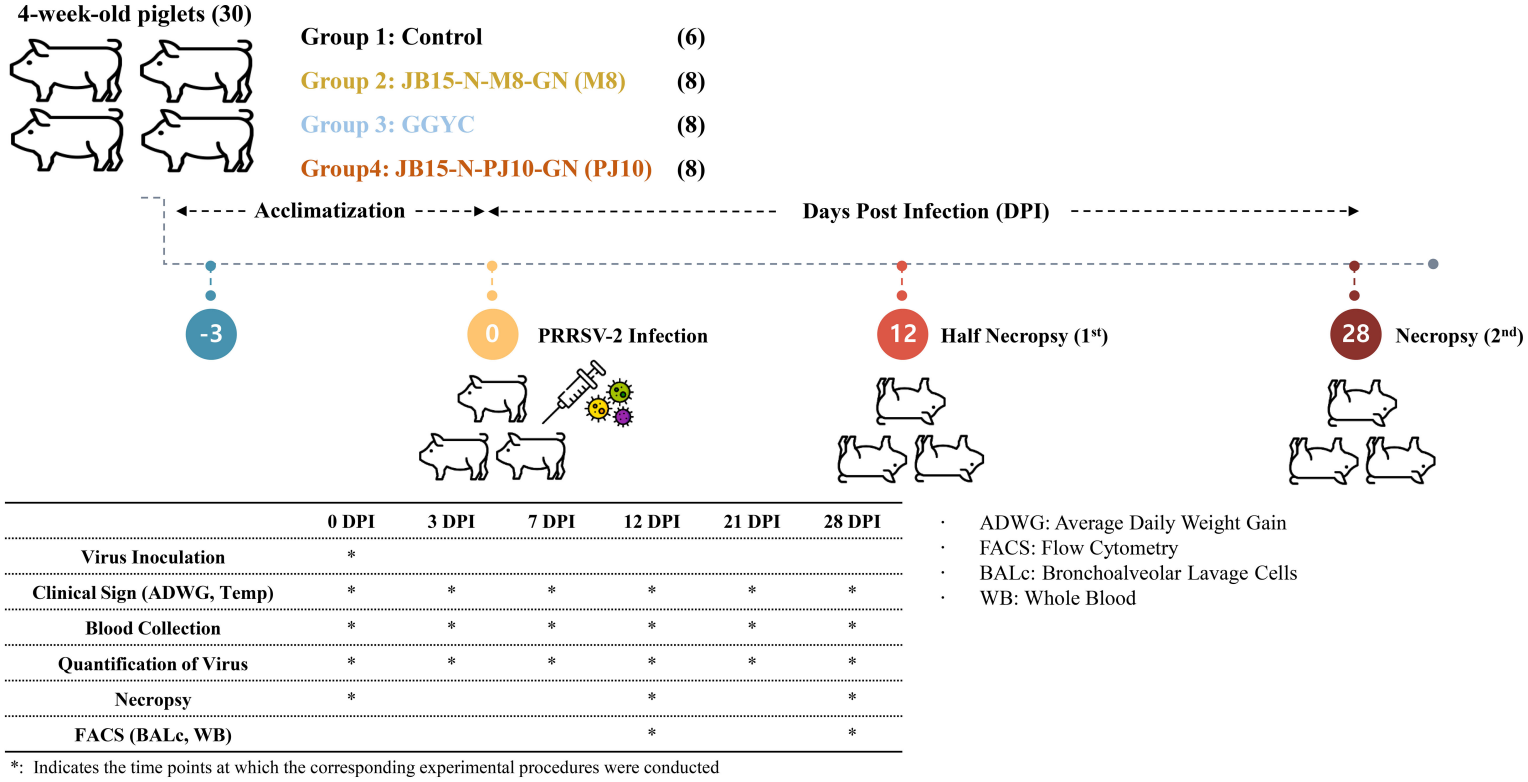
Experimental design overview. Thirty 4-week-old piglets from a PRRSV- and PCV2-seronegative farm were randomly divided into four groups. After a 3-day acclimatization, infected groups received 2 mL of PRRSV-2 strain via intramuscular inoculation. Daily monitoring, including temperature checks and clinical observation, was conducted. Blood samples were collected at various dpi intervals, and body weights were recorded. Euthanasia was performed at 12 and 28 dpi. Tissue samples were collected for histopathological examination, and bronchoalveolar lavage cells were isolated for further analysis.

Thirty 4-week-old piglets obtained from a PRRSV- and PCV2-seronegative farm were randomly assigned to four groups. An overview of the animal experiment is depicted in **Figure 1**. Following a 3-day acclimatization, the infected groups were administered 2 mL of PRRSV-2 strain diluted in sterile PBS. Eight pigs in each group were intramuscularly inoculated with M8, GGYC45, and PJ10 at a concentration of 1 x 10^3^ TCID_50_/mL, respectively. Control pigs (n=6) remained uninfected. Daily monitoring of all pigs was conducted three days before inoculation and continued until the end of the experiment. The pigs were fed twice daily, and monitoring, including temperature measurement, was conducted during feeding times. Blood collection and nasal swab samplings were conducted at 0, 3, 7, 12, 21, and 28 dpi. Body weights of all pigs were recorded, while the average daily weight gain (ADWG) of euthanized pigs was assessed at 12 and 28 dpi. To elucidate the pathogenicity of Korean PRRSV-2, half of the animals in each group were humanely euthanized at 12 dpi, and the remaining half at 28 dpi for necropsy. Euthanasia was performed via electrocution following an intramuscular injection of 2 mL of azaperone (40 mg/mL, StressGuard^®^, Dong Bang Inc., Republic of Korea). The pigs were allowed to reach adequate sedation approximately 10–15 minutes post-injection. Once adequate sedation was confirmed, electrocution was performed using 60 Hz alternating current applied across the brain and heart for 5–10 seconds to ensure immediate loss of consciousness. Exsanguination was performed immediately following electrocution to complete the euthanasia process.

Two pigs infected with PJ10 died during the experiment due to severe clinical signs, high fever, and reduced body weight from the PJ10 group. The experiment for the PJ10 group was terminated at 12 dpi as the pigs were deemed to be in a moribund state. Post-euthanasia, respiratory organs, including lungs, trachea, and bronchi, were aseptically excised and rinsed with 75 mL of sterile PBS. Bronchoalveolar lavage cells (BAL cells) were isolated from the collected lung lavage fluid by centrifugation at 1000 ×g at room temperature for 10 minutes. BAL cells were stored in tubes containing cell banker (CELLBANKER^®^ Cell Freezing Media, Amsbio, UK) and immediately frozen at -80°C for subsequent RNA extraction and immune analysis. Lung and brain tissues were preserved in 10% neutral-buffered formalin for histopathological examination.

The animal experiment protocol was approved by the Jeonbuk National University Institutional Animal Care and Use Committee (JBNU 2021-095) and performed according to the guidelines and regulations detailed by the committee.

### 2.3 Quantification of viral load

Serum viremia levels were assessed at designated days, and viral loads in the tissues (Nasal swab, lung, and brain) were quantified in euthanized pigs at 12 and 28 dpi. Viral RNA extraction from serum and tissues was conducted using the MagMAX viral RNA isolation kit (Ambion; Applied Biosystems, Life Technologies, Inc.). The viral loads were quantified through real-time reverse transcription-polymerase chain reaction (RT–qPCR) utilizing the Prime-Q PCV2, PRRSV Detection Kit (Genet Bio, Republic of Korea).

### 2.4 Anti-PRRSV specific antibody detection

Serum samples from all groups were assessed for anti-PRRSV-specific antibody (IgG) using a commercially available ELISA kit (PRRS Ab ELISA 4.0; Bionote Inc., South Korea) following the manufacturer’s instructions. Serum samples exhibiting an S/P ratio (the ratio of the net optical density of the test samples to the net optical density of the positive controls) ≥ 0.4 were classified as positive for PRRSV-specific antibody, as previously described (26, 28).

### 2.5 Cytokine immunoassay

Cytokine levels in the serum from all groups were evaluated utilizing a commercially available porcine-specific ProcartaPlex™ Multiplex Immunoassay (ThermoFisher Scientific, Vienna-1030, Austria) following the manufacturer’s instructions. The concentration of each cytokine was determined by analyzing the samples on the Luminex^®^ 200TM system (Luminex Corporation, Austin, TX, USA). The provided standards in the kit were utilized. The machine underwent verification and calibration using a Luminex^®^ 100/200TM verification kit and a Luminex^®^ 100/200™ calibration kit following the manufacturer’s guidelines (26).

### 2.6 Histopathological evaluations

The lungs and brains of necropsied pigs in all experimental groups performed a pathologically assessment at 12 and 28 dpi. Macroscopic and microscopic lung lesions were evaluated following established protocols (23, 26, 41). A macroscopic lung lesion was scored for the percentage of lungs affected by pneumonia in the right lung lobes using a predefined evaluation system. Microscopic lung lesions from three distinct lung lobes were graded on a scale from 0 to 3, reflecting the absence of lesions or mild interstitial pneumonia, moderate-multifocal interstitial pneumonia, moderate diffuse interstitial pneumonia, and severe interstitial pneumonia, respectively. Microscopic brain lesions were similarly scored on a scale from 0 to 3, indicating the absence of lesions or minimal-multifocal perivascular inflammatory cell infiltration, mild-multifocal perivascular inflammatory cell infiltration, moderate-multifocal perivascular inflammatory cell infiltration, and severe multifocal perivascular inflammatory cell infiltration.

Immunohistochemistry (IHC) was employed to detect PRRSV-specific antigens in infected brain tissue. For IHC staining, tissue embedded on a glass slide was treated with an endogenous peroxidase inhibitory reagent (3% H_2_O_2_ in phosphate-buffered saline (PBS), pH 7.2) at room temperature for 10 minutes, followed by incubation with 0.05% protease type XIV (Sigma, USA) at 37°C for 20 minutes for antigen retrieval. Subsequently, the primary antibody (monoclonal mouse anti-PRRSV, SDOW17) antibody, RTI, LLC, USA) was applied at a dilution ratio of 1:1000 and incubated at 37°C for 1 hour. Next, the secondary antibody (EnVision™/HRP, Rabbit/Mouse (EVN) reagent, DAKO, Denmark) was added and incubated at 37°C for 40 minutes. The IHC analysis was then conducted using 3,3-diaminobenzidine tetrahydrochloride (DAB) staining (DAKO, Denmark).

### 2.7 Flow cytometry

Flow cytometry was utilized to analyze cell surface, intracellular, and intranuclear staining for each cell type. Single-cell suspensions underwent cell surface staining by incubating with specific antibodies listed in **Table 1** on ice for 30 minutes, followed by three washes with FACS buffer (3% FBS in PBS). NK cells, DCs, and macrophages (MǾs) were analyzed post-cell surface staining, while other T cell subsets required intranuclear and intracellular staining. BAL cells were preferentially subjected to cell surface staining for the CD163 surface marker, followed by the identification of DCs and MǾs in the BAL cell population using staining and gating strategies (42, 43).

**Table 1:** Cell population and antibodies for FACS.

Cell population	Antibody	Note
NK cell	CD3 FITC	Cell surface staining
	CD8 PE	
	CD335 IgG1	
	APC IgG1	
Helper T cell (Th1, Th17)	CD4 PE	Need stimulation Intracellular staining
	IFN-γ PerCP Cy5.5	
	IL17 APC	
	CD8 FITC	
Cytotoxic T cell (CTL)	CD3 FITC	Need stimulation Intracellular staining
	CD8 PE	
	TCR IgG1	
	APC IgG1	
	IFN-γ PerCP Cy5.5	
Dendritic cell (DC)	CD163 IgG1	Only for BALc
	FITC IgG1	
	MHCII IgG2α	
	PerCP IgG2α	
	CD172α PE	
T cell subsets (CD3, CD4, CD8)	CD3 FITC	Cell surface staining
	CD4 PerCP	
	CD8 PE	

Cell surface staining: No need for fixation, permeabilization buffer.

Intracellular/nuclear staining: Need fixation, permeabilization buffer.

Five functionally and phenotypically defined subpopulations were distinguished from the MHCII^+^ cell population via CD163 and CD172α surface markers, including alveolar macrophages (AMs, CD172α^+^/CD163^high^), monocyte-derived macrophages (moMǾs, CD172α^+^/CD163^int^), monocyte-derived dendritic cells (moDCs, CD172α^+^/CD163^low^), conventional dendritic cells 1 (cDC1s, CD172α^+^/CD163^-^), and conventional dendritic cells 2 (cDC2s, CD172α^-^/CD163^-^) (26, 27, 43).

Two subsets of NK cells were analyzed based on NKp46 marker expression, classified as NKp46^+^ and NKp46^-^ NK cells (44, 45). CD3^-^ lymphocytes were preferentially sorted and further analyzed for CD8α and NKp46 expression. Through this process, two populations were found in FACS analysis. NK cells were classified as NKp46^+^ (CD8^+^/NKp46^+^/CD3^-^) and NKp46^−^ (CD8^-^/NKp46^+^/CD3^-^) NK cells and both of these cells were CD8α^+^. In addition, the T-cell subsets subjected to intracellular staining were stained according to previous studies (26). CD3- lymphocytes were sorted and further analyzed for CD8α and NKp46 expression, resulting in the identification of NK cells as NKp46^+^ (CD8^+^/NKp46^+^/CD3^-^) and NKp46− (CD8^-^/NKp46^+^/CD3^-^) NK cells, both expressing CD8α^+^. Intracellular staining was performed for T cell subsets such as cytotoxic T cells (CTLs).

Stained cells were suspended in FACS buffer and analyzed using an Accuri C6 flow cytometer (BD Accuri™ C6 Plus, BD Biosciences, MD, USA). Data analysis was performed on 100,000 events using FLOWJO version 10.6.1 (LLC, Ashland, OR 97520), excluding unstained doublets and irrelevant populations. Auto compensation was set based on color control and isotype control stains. FACS data are presented as percentages of all cell subsets.

### 2.8 Quantitative real-time polymerase chain reaction of BAL cells

RNA was extracted using the GeneAll^®^ RiboEx™ kit (GeneAll, Republic of Korea) following the manufacturer’s instructions. The concentration and purity of the isolated RNA were determined spectrophotometrically using a Nanodrop (Biospec Nano, Shimadzu, Japan), with samples meeting the 260/280 ratio criteria. Subsequently, cDNA was synthesized utilizing the WizScript™ cDNA Synthesis Kit - High Capacity (Wizbio, Republic of Korea) following the manufacturer’s protocol. Quantitative reverse-transcription PCR (RT-qPCR) was conducted using the extracted samples, employing primers detailed in **Table 2**.

**Table 2:** Primers used for real-time quantitative RT-PCR.

Target	Target gene	Primer	Sequence (5’ -> 3’)
Immune Checkpoint Molecule	T cell Exhaustion	PD1F	AGC CCA AGC ACT TCA TCC TC
		PD1R	TGT GGA AGT CTC GTC CGT TG
		PDL1F	GTG GAA AAA TGT GGC AGC CG
		PDL1R	TGC TTA GCC CTG ACG AAC TC
		CTLA4F	TCT TCA TCC CTG TCT TCT CCA AA
		CTLA4R	GCA GAC CCA TAC TCA CAC ACA AA
		TIM3F	TTC GAC GGG AGC AGT AAA GC
		TIM3R	AGG GCA GGA CAC AGT CAA AG
		LAG3F	CTC CTC CTG CTC CTT TTG GTT
		LAG3R	CAG CTC CCC AGT CTT GCT CT
		IDO1F	GGC ACT TGA TTG GTG GTC TC
		IDO1R	GCA ATC CAA GCA TCG TAA GG

### 2.9 Data analysis

Graphical representations of data were generated using GraphPad Prism 9.00 (GraphPad, USA), and statistical analyses were conducted using SPSS Advanced Statistics 17.0 software (SPSS, USA). Two-way ANOVA with Tukey’s multiple comparison tests was employed to assess the significance of variability within animal experimental groups for clinical data, including viremia, viral load, temperature, and anti-PRRSV antibodies. For comparisons of average daily weight gain (ADWG), phenotypes of various cell subsets, and mRNA expression, a nonparametric one-way ANOVA (Kruskal–Wallis test) was utilized. Statistically significant differences are denoted by asterisks and different letters over the bars.

## 3 Results

### 3.1 Clinical and pathological features of Korean PRRSV-2 infected pigs

Genetically distinct Korean PRRSV-2 induced various clinical and pathological symptoms. Especially, the PJ10, a Korean Lineage B (LKB) PRRSV-2 strain, induced severe clinical symptoms, including severe weight loss, respiratory distress, and high fever, resulting in significant morbidity and mortality.

#### 3.1.1 Mortality

**Figure 2:**
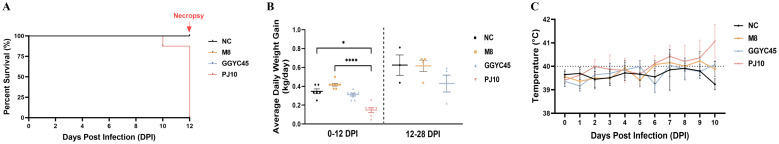
Clinical and pathological features of Korean PRRSV-2 infected pigs. **(A)** Survival curve of pigs infected with the PRRSV-2 strain PJ10, a Korean Lineage B (LKB) strain. Infected pigs exhibited severe clinical signs, including significant weight loss, respiratory distress, and persistent high fever, which ultimately led to sudden death. **(B)** Average daily weight gain (ADWG) comparison among pig groups challenged with genetically distinct PRRSV-2 strains M8, GGYC45, and PJ10 at 12 and 28 days post-infection (dpi). **(C)** Body temperature profiles of pigs infected with different PRRSV-2 strains, highlighting elevated temperatures exceeding 40°C observed in the PJ10-infected group at 6-10 dpi. The bars represent the mean, and the error bars represent the standard error of the mean (SEM). Bars with asterisks (*) indicate significantly different values (*indicates *p* < 0.05, ****indicates *p*<0.0005).

Notably, two pigs in the PJ10-infected group, belonging to Korean Lineage B (LKB), died at 10 and 12 dpi. The other pigs also displayed severe weight loss, respiratory signs, high fever, and severe nervous signs such as ataxia (**Figure 2A**). To comply with animal ethics, the remaining pigs in the PJ10 infected group were humanely euthanized at 12 dpi due to the severity of clinical symptoms. In contrast to PJ10-infected pigs, the M8- and GGYC45-infected pigs did not observe death until the end of the experiment.

#### 3.1.2 Weight gain

The average daily weight gain (ADWG) of the PJ10-infected group was significantly lower than that of the control and other infected groups at 12 dpi (*p < 0.05* and *p < 0.0005*, respectively). Specifically, while the ADWG per control pig was 0.347 kg, the ADWG in the infected groups was recorded at 0.417, 0.313, and 0.149 kg for M8, GGYC45, and PJ10 at 12 dpi, respectively. At 28 dpi, the ADWG in the M8-, GGYC45-infected pigs increased by approximately 0.11 kg and 0.07 kg, respectively; however, both remained significantly lower than the control pigs (**Figure 2B**).

#### 3.1.3 Body temperature

Pigs infected with PJ10 exhibited elevated body temperatures compared to other infected groups. The M8- and GGYC45-infected pigs showed relatively high fever (around 40°C), however, the PJ-10-infected pigs observed exceeding 40°C observed at 6–10 dpi (**Figure 2C**).

#### 3.1.4 Viremia and virus shedding

**Figure 3:**
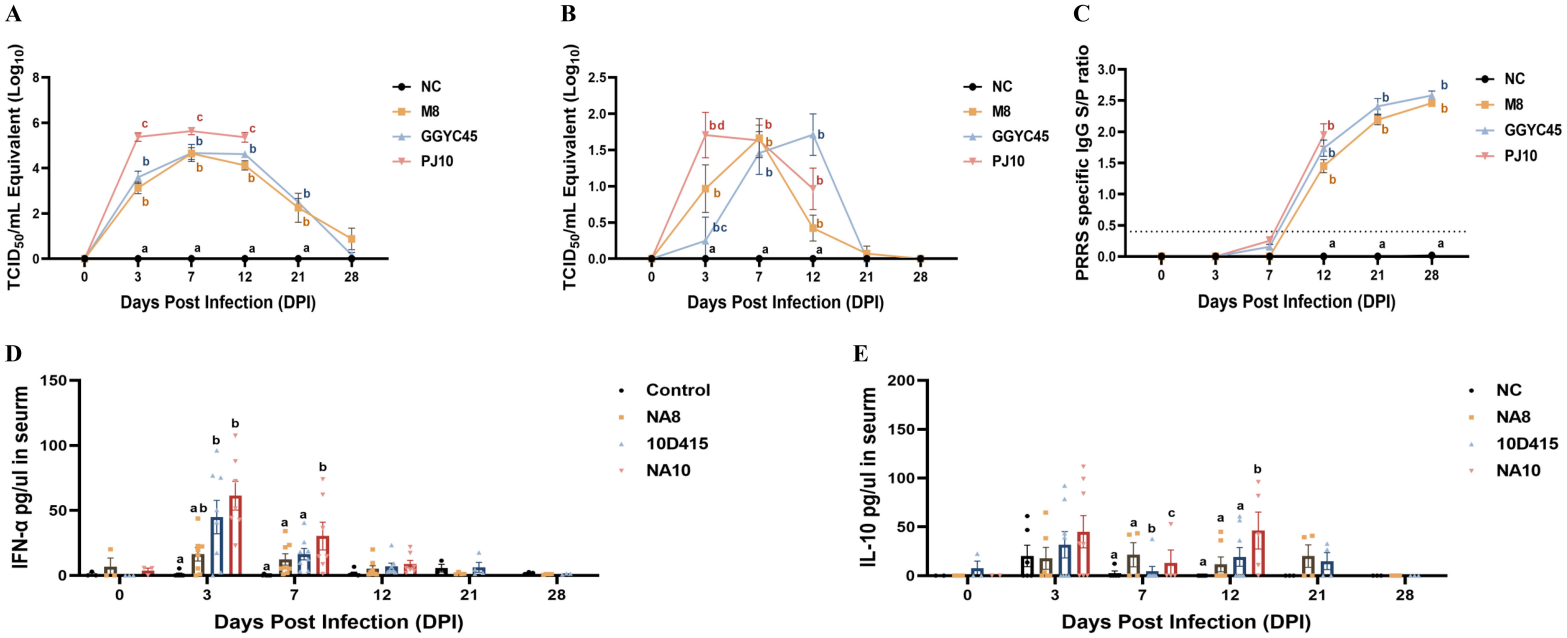
Pathogenicity of Korean PRRSV-2: viremia, nasal shedding, and antibody, cytokine response. **(A)** Mean peak virus titers in serum (viremia) measured as TCID _50_/mL at 3 dpi were recorded as 10^3.12^, 10^3.59^, and 10^5.37^ in the M8, GGYC45, and PJ10 infected groups, respectively. **(B)** Mean peak virus titers in nasal virus shedding measured as TCID _50_/mL were detected at 3, 7, and 12 dpi with values of 10^0.69^, 10^0.22^, and 10^1.22^ in the M8, GGYC45, and PJ10 infected groups at 3dpi, respectively. **(C)** PRRSV-specific antibody (IgG) levels measured by ELISA at 12 dpi with mean peaks of the S/P ratio were 1.448, 1.736, and 1.948 in the M8, GGYC45, and PJ10 infected groups, respectively. **(D)** Serum cytokine protein levels comparison between uninfected and infected groups showing significant (*p* < 0.0005) induction of interferon-a (IFN-a), a proinflammatory cytokine, in PJ10 infected pigs at 3 and 7 dpi. **(E)** Significant (*p* < 0.0005) induction of interleukin-10 (IL-10), an anti-inflammatory cytokine, at 7 and 14 dpi in infected groups compared to uninfected controls. The error bars represent standard deviations.

The PJ10-infected group demonstrated the highest viremia and nasal swab shedding between 3 and 12 dpi. Conversely, the control group maintained an uninfected status throughout the experiment. The mean peak of viral titer in serum (viremia) was recorded as 10^3.12^, 10^3.59^, and 10^5.37^ TCID_50_/mL in the M8, GGYC45, and PJ10 infected groups at 3 dpi, respectively. While the PJ10-infected group showed a similarly high level of viremia and died until 12 dpi, the viremia of the other two groups was observed to increase until 7 dpi and then decreased thereafter. In the nasal virus shedding results, the PJ10 showed the highest virus level (10^1.22^ TCID_50_/mL) at 3 dpi, while M8 showed the highest virus titre at 7 dpi, and GGYC45 showed the highest virus titre at 12 dpi. (**Figures 3A, B**).

#### 3.1.5 PRRSV-specific antibody

PRRSV-specific antibody (IgG) levels were measured by commercialized ELISA, with the control group showing no detectable PRRSV-specific IgG. There were no significant differences among the challenged group, however, the PJ10-infected pigs showed robust antibody responses compared to other strains. The M8, GGYC45, and PJ10 exhibited mean peaks of the S/P ratio of 1.448, 1.736, and 1.948 at 12 dpi, respectively, with antibody levels increasing up to 28 dpi (**Figure 3C**).

#### 3.1.6 Cytokine

Serum cytokine protein levels were compared after genetically distinct PRRSV-2 infections. Interferon-α (IFN-α), a proinflammatory cytokine, was significantly induced (*p < 0.005*) in LKB-infected groups (GGYC45 and PJ10) at 3 and 7 dpi. Especially, the PJ10-infected groups showed high levels of IFN-α at the early time of infection (**Figure 3D**). Interestingly, the level of interleukin-10 (IL-10), an anti-inflammatory cytokine, was also slightly or significantly elevated in the LKB-infected (GGYC45 and PJ10) groups until 12 dpi (**Figure 3E**).

### 3.2 Comprehensive analysis of respiratory pathology in PRRSV-2 infection: gross pathology, histopathology, and lung viral load

#### 3.2.1 Macroscopic and microscopic pathology

**Figure 4:**
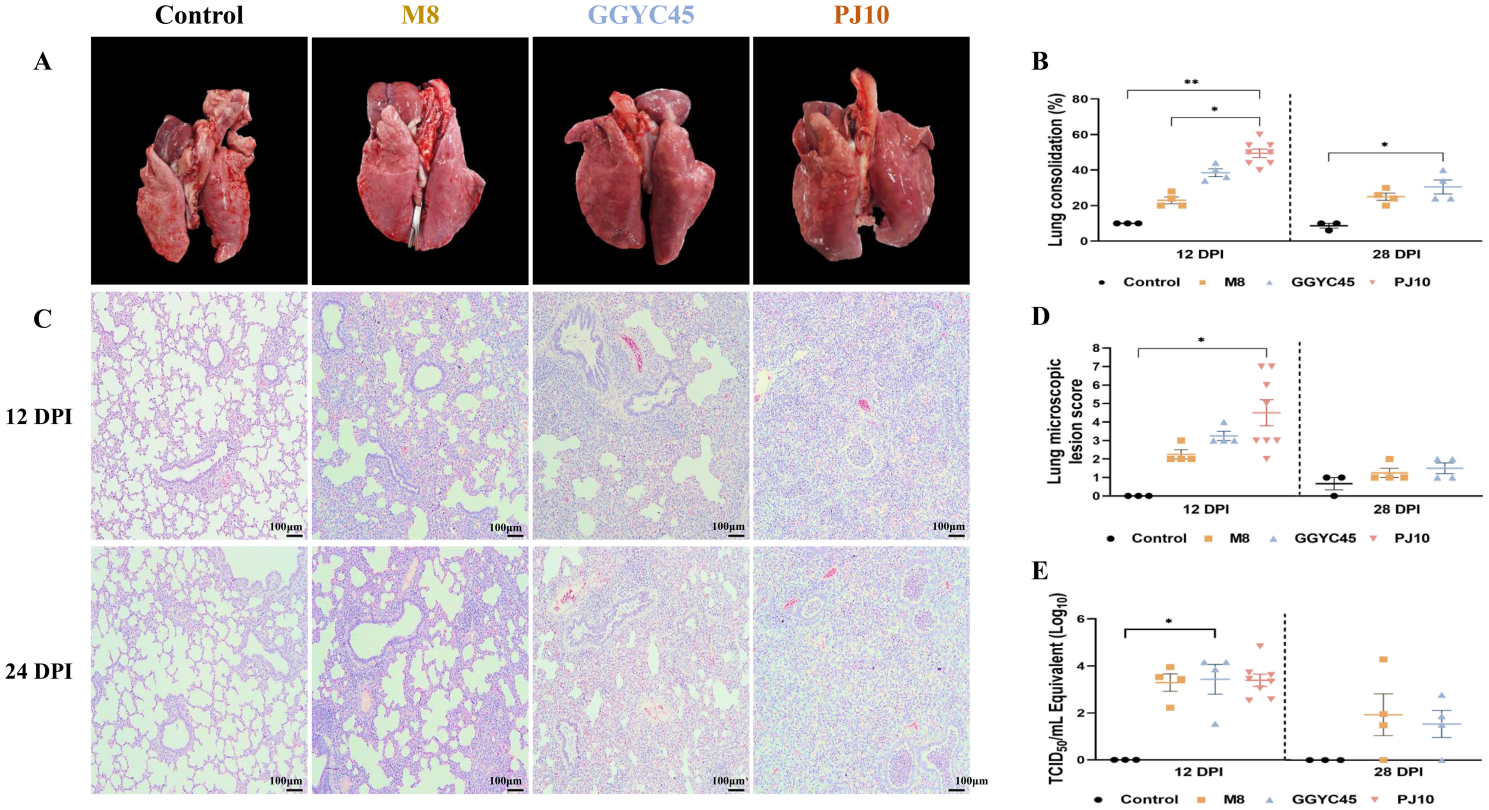
Comprehensive analysis of respiratory pathology in PRRSV-2 infection: gross pathology, histopathology, and lung viral load **(A)** Gross examination of lung tissues from control and PRRSV-infected pigs at 12 and 28 days post-infection (dpi). Pigs infected with GGYC45 and PJ10 strains exhibited severe lung consolidation compared to the M8-infected and control group. **(B)** Average lung lesion scores indicated significantly higher scores in infected groups compared to the control group, with the PJ10 infected group showing the highest scores among the infected groups. **(C)** Histopathological analysis observed interstitial pneumonia with alveolar wall thickening and inflammatory cell infiltration in PRRSV-infected groups at 12 dpi (Bar =100μm). **(D)** Microscopic lung lesion scores at 12 dpi demonstrate significantly higher levels in the PJ10 infected group than the other infected groups, with a subsequent decrease in inflammatory response by 28 dpi. **(E)** Viral titration in lung tissues showed slightly higher mean titers in the GGYC45 and PJ10 infected groups compared to the M8-infected group at 12 dpi The bars represent the mean and the error bars represent the standard error of the mean (SEM). Bars with asterisks (*) indicate significantly different values (*indicates *p* < 0.05, ****indicates *p*<0.0005).

The PJ10-infected group exhibited more severe gross lesions, characterized such as diffuse dark-red consolidation and firmness, than the M8- and GGYC45-infected groups. Microscopically, the PJ10-infected lungs displayed more extensive interstitial thickening, alveolar septal infiltration, and mononuclear cell accumulation, consistent with severe diffuse interstitial pneumonia, whereas the M8 and GGYC45 groups showed mild to moderate multifocal lesions. In contrast, no macroscopic (lung-gross) lesions were observed in the negative control groups (**Figure 4A**). The average lung lesion (Lung consolidation) score was significantly higher (*p < 0.05*) in the LKB-infected group than in the M8-infected group. Notably, among the infected groups, the PJ10-infected group demonstrated significantly higher levels than the other strains (**Figure 4B**). Histopathological evaluations revealed that the PJ10-infected group displayed mild to severe multifocal/diffuse interstitial pneumonia with alveolar wall thickening due to pneumocyte proliferation, along with inflammatory cell infiltration in the infected groups at 12 dpi (**Figure 4C**). The lung microscopic lesions in the M8- and GGYC45-infected groups showed a slight increase due to PRRSV infection. Specifically, the highest microscopic lung lesion was observed at 12 dpi in the PJ10-infected group, and the lung lesion score of pigs was also significantly *(p < 0.05*) higher. Subsequently, the inflammatory cell infiltration and inflammatory response induced by PRRSV infection gradually decreased until 28 dpi (**Figure 4D**).

#### 3.2.2 Lung viral load

Viral load was quantified to reveal distinct characteristics of viral replication in the lungs. The groups infected with the PJ10 and GGYC45 strains demonstrated slightly elevated mean titer (10^3.43^ and 10^3.38^ TCID_50_/mL, respectively) compared to those observed in the M8-infected group at 12 dpi. “At 28 dpi, the lung viral loads of the M8- and GGYC45-infected groups were analyzed as 10^1.69^ and 10^1^ TCID_50_/mL, respectively; however, no significant differences were observed between infected groups (**Figure 4E**).

### 3.3 Comprehensive analysis of neurological pathology in PRRSV-2 infection: histopathology and brain viral load

#### 3.3.1 Histopathological examination

**Figure 5:**
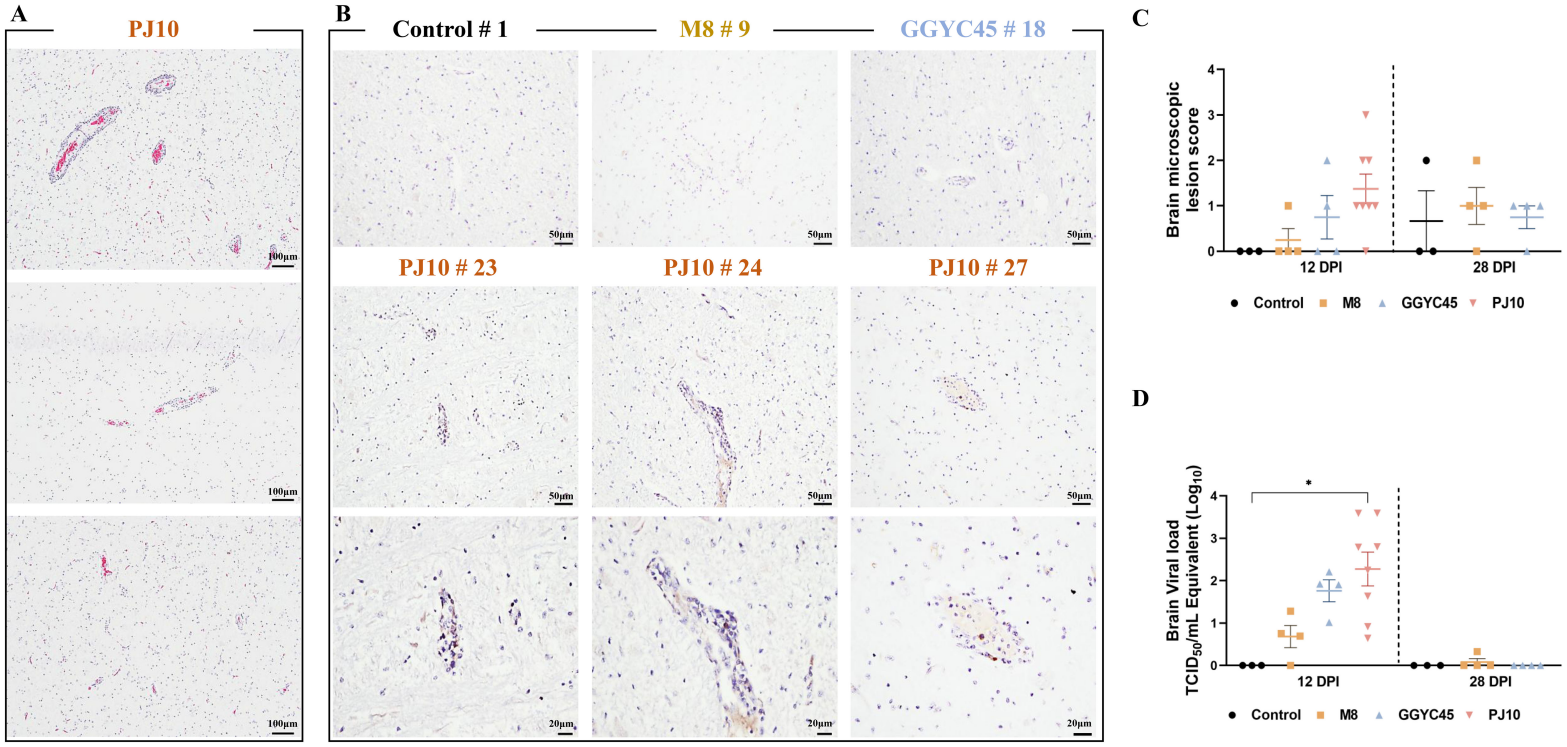
Comparison of brain viral load and histopathological findings in PRRSV-infected pigs H&E staining and IHC were performed to observe the histopathological features of brain tissue sections obtained from samples collected at 12 dpi after PJ10 infection **(A)** H&E assay reveals mild levels of perivascular inflammatory cell infiltration and infiltrated glial nodules in the Virchow-Robin space surrounding the brain in PJ10-infected pigs. Distinct inflammatory cell-infiltrated lesions were exclusively observed in the PJ10 infected group, with positive reactions confirmed in the cytoplasm of cells located within some perivascular inflammatory cell infiltration sites (Bar=100μm). **(B)** Three PJ10-infected pigs exhibit a PRRSV-specific IHC reaction in the cytoplasm located within the perivascular inflammatory cell-infiltrated lesion (Bar =50μm, 20μm). **(C)** Microscopic lesion scores indicate a slight increase in the infected group at 12 and 28 dpi. The bars represent the mean and the error bars represent the standard error of the mean (SEM). **(D)** Mean titers of the PJ10-infected group (10^2.27^ TCID _50_/mL) show a significant increase compared to other infected groups (10^0.68^ and 10^1.76^ TCID _50_/mL, respectively). Bars with asterisks (*) indicate significantly different values (*indicates *p* < 0.05).

To investigate PRRSV-induced brain lesions, brain tissues collected at 12 dpi and 28 dpi were analyzed. However, pathological findings associated with PRRSV infection were not observed in any groups except for the PJ10-infected group. Therefore, brain tissue analysis was performed on the PJ10-infected group at 12 dpi. Histopathological analysis using the H&E assay unveiled mild levels of perivascular inflammatory cell infiltration and infiltrated glial nodules in the Virchow-Robin space surrounding the brain in PJ10-infected pigs. Notably, distinctive inflammatory cell-infiltrated lesions attributed to PRRSV infection were exclusively observed in the PJ10-infected group, with positive reactions confirmed in the cytoplasm of cells located within some perivascular inflammatory cell infiltration sites (**Figure 5A**). Immunohistopathological evaluation (IHC) of the brain revealed no lesions associated with PRRSV infection in any group except for the PJ10-infected group. Remarkably, three PJ10-infected pigs exhibited a positive IHC reaction in the cytoplasm within the perivascular inflammatory cell-infiltrated lesion (**Figure 5B**). Microscopic examination revealed a slightly higher lesion score in the brains of the PJ10-infected group at 12 dpi (**Figure 5C**).

#### 3.3.2 Brain viral load

The PJ10-infected group exhibited significantly (*p < 0.05*) higher mean titers (10^2.27^ TCID_50_/mL) in the brain compared to the other infected groups (10^0.68^ and 10^1.76^ TCID_50_/mL, M8 and GGYC45, respectively) (**Figure 5D**).

### 3.4 Temporal dynamics of immune cell populations in PRRSV-2 infected pigs: comprehensive analysis of monocytes, T lymphocytes, and NK Cells in BAL cells

#### 3.4.1 DC/macrophage networks

**Figure 6:**
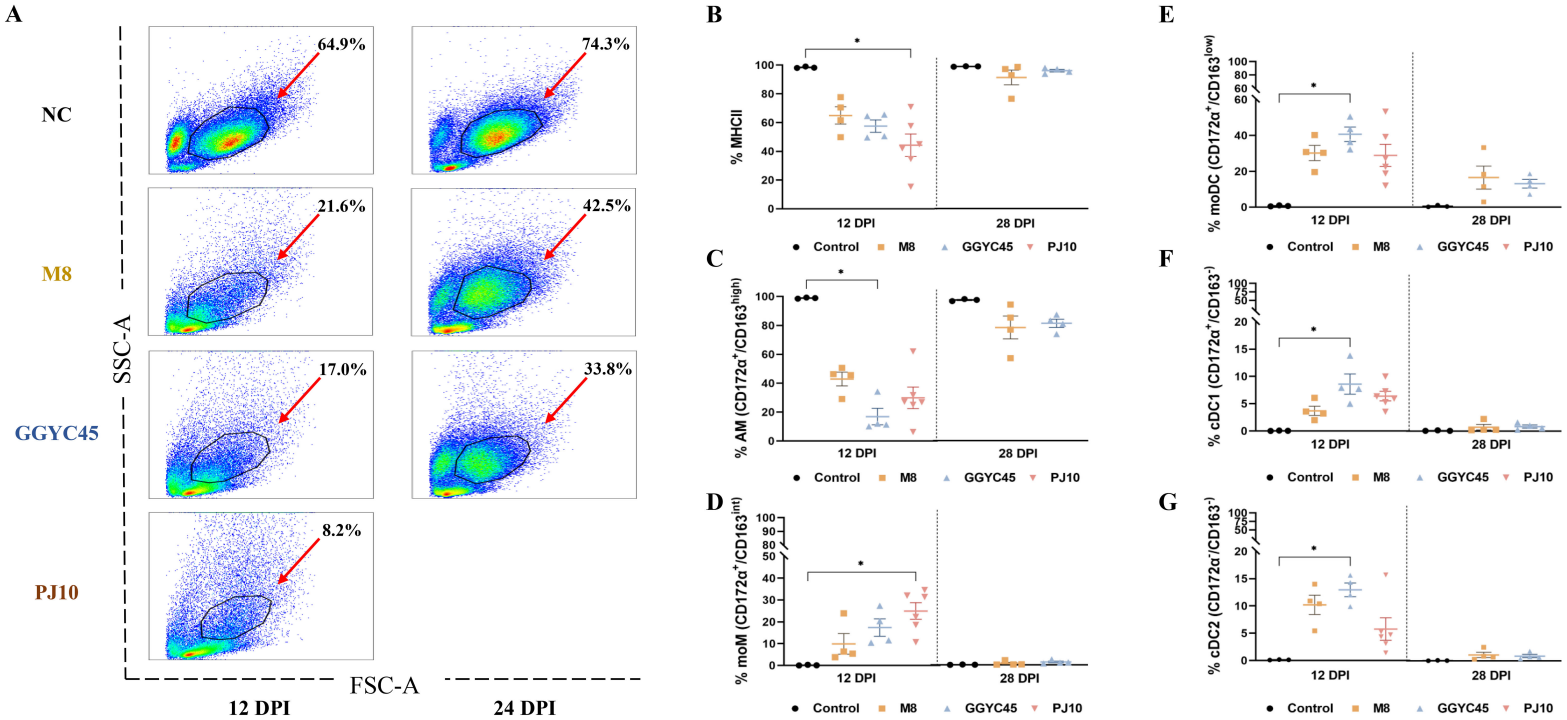
The dynamics of the lung immune cell population are altered after genetically unique PRRSV infection in BAL cells: Monocytes **(A)** Flow cytometric analysis of bronchoalveolar lavage (BAL) cells. **(A)** Distribution of cell populations in BAL fluid across experimental groups. **(B)** Changes in major histocompatibility complex class II positive (MHCII) cell populations at 12 days post-infection (dpi). **(C)** Percentage of alveolar macrophages (AMS) (CD172a/CD163high) among MHCII cells, indicating a significant decrease in infected groups at 12 dpi, particularly noticeable in GGYC45-infected groups (*p*<0.05). **(D)** Increase in monocyte-derived macrophages (moMs, CD172a/CD163int), notably higher in PJ10-infected group at 12 dpi (*p* < 0.05). **(E-G)** Significant increase in monocyte-derived dendritic cells (moDCs) and conventional dendritic cells (DC1/2) at 12 dpi, with the GGYC45-infected group exhibiting the highest rise compared to other infected groups (*p*<0.05). By 28 dpi a slight decrease in these cell populations is observed, approaching levels similar to the control group. The bars represent the mean and the error bars represent the standard error of the mean (SEM). Bars with asterisks (*) indicate significantly different values (*indicates *p* < 0.05, **indicates *p*<0.005).

In bronchoalveolar lavage (BAL) cell flow cytometry, the control group exhibited a stable subset of the cell population. Monocyte populations constituted 64.9% in the control group; however, at 12 dpi, they were significantly reduced in the infected groups, with percentages of 21.6% in the M8-infected group, 17.0% in the GGYC-infected group, and 8.2% in the PJ10-infected group. By 28 dpi, the mixture of monocyte, neutrophils, and lymphocyte populations in the M8-infected group and GGYC45-infected group showed partial recovery, reaching approximately 21% and 16%, respectively, although lower than the control group (**Figure 6A**).

#### 3.4.2 Monocytes

Following an initial evaluation of the overall monocyte population, a comprehensive analysis was performed on the individual monocyte subpopulations. The population of major histocompatibility complex class II positive (MHCII^+^) cells decreased at 12 dpi in the infected groups. The average percentage of MHCII+ cells in the control group was 97.9%, while in the M8-infected group, GGYC45-infected group, and PJ10-infected group, it was 62.9%, 56.2%, and 51.2%, respectively. Although no significant differences were observed between the infected groups, it was noted that the PJ10-infected group showed a greater reduction compared to the other infected groups (**Figure 6B**). The percentages of alveolar macrophages (AMs; CD172α^+^/CD163^high^), constituting the major MHCII^+^ cells, were significantly decreased in all infected groups at 12 dpi. The average percentage of AM in the control group was 93.5%, while in the M8-infected group, GGYC45-infected group, and PJ10-infected group, it was 20.8%, 5.6%, and 10.1%, respectively. A significant difference (*p < 0.05*) was observed in the GGYC45-infected group. By 28 dpi, the decreased AM ratio had been restored to approximately 50% (**Figure 6C**).

#### 3.4.3 Monocyte-derived cell populations

In contrast to MHCII^+^ cells and AMs, the proportion of the monocyte-derived macrophage (moMs; CD172α^+^/CD163^int^) infiltrating the lung was significantly increased in PRRSV-infected groups. The average percentage of moMs in the control group was 0.5%, while in the M8-infected group, GGYC45-infected group, and PJ10-infected group, it was 5.6%, 9.6%, and 13.1%, respectively. Notably, a significant (*p < 0.05*) influx of moMs was observed in the PJ10-infected group at 12 dpi (**Figure 6D**). Similar to the moM findings, monocyte-derived dendritic cells (moDCs; CD172α^+^/CD163^low^) and conventional DC1/2 (cDC1; CD172α^+^/CD163^-^, cDC2; CD172α^-^/CD163^-^) also significantly increased in the infected group at 12 dpi (*p < 0.05*). Notably, the GGYC45-infected group showed a significant (*p < 0.05*) influx of moDCs and cDC1 compared to the other infected groups. Although not statistically significant, a similar trend was observed in the PJ10-infected group, resembling the GGYC45-infected group. (**Figures 6E–G**).

#### 3.4.4 T lymphocyte subsets

**Figure 7:**
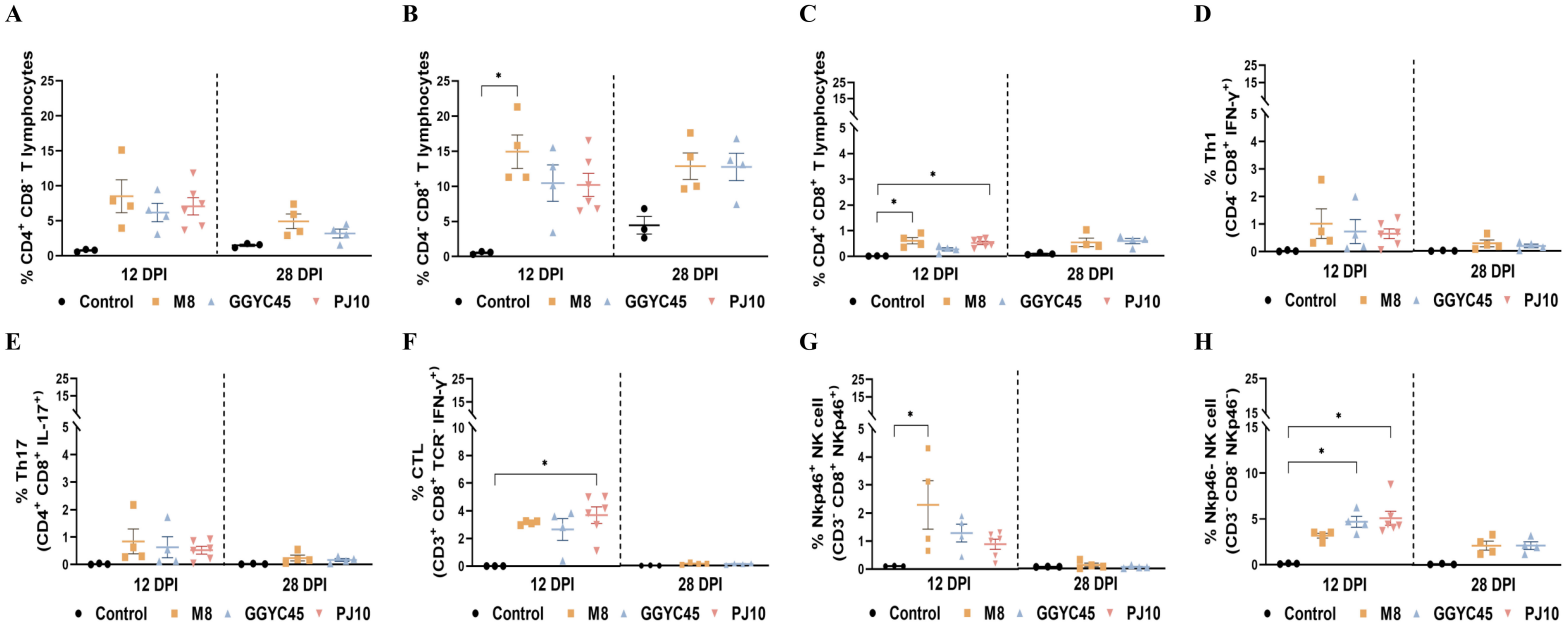
The dynamics of the lung immune cell population are altered after genetically unique PRRSV infection in BAL cells: T Cells, NK cells **(A)** Analysis of CD3+CD4+ T lymphocytes showing a slight increase at 12 days post-infection (dpi) compared to control levels. **(B)** Significant increase (*p* < 0.05) in CD3+*CD8+ T lymphocytes, especially in the M8-infected group, persisting up to 28 dpi. **(C)** Significant (*p* < 0.05) increase in CD3+CD4+CD8+ T lymphocytes, particularly in the M8 and PJ10 infected groups. **(D)** Th1 (IFN-y+/CD4/CD8+) and **(E)** Th17 (IL-17/CD4+/CD8+) responses slightly higher than controls at 12 dpi, with no significant difference among infected groups. **(F)** Significantly (*p*<0.05) higher frequency of cytotoxic T lymphocytes (CTL) (IFN-y+/CD3+/TCR-/CD8+) in BAL cells of PJ10 infected group at 12 dpi, returning to normal at 28 dpi. **(G)** Slightly higher percentages of NKp46+ (CD8+/Nkp46/CD3-) NK cells in infected group, notably significantly (*p*<0.05) higher in M8 infected group at 12 dpi. **(H)** Significant (*p*<0.05) increase in percentages of NKp46- (CD8-/Nkp46+/CD3-) NK cells in BAL cell population of infected pigs, especially in PJ10 infected group at 12 dpi. The bars represent the mean and the error bars represent the standard error of the mean (SEM). Bars with asterisks (*) indicate significantly different values (*indicates *p* < 0.05).

The frequencies of T lymphocyte subsets, including CD3^+^CD4^+^, CD3^+^CD8^+^, and CD3^+^CD4^+^CD8^+^ populations, in BAL cells were analyzed. Regardless of the PRRSV strains, all infected groups exhibited an increase in these subpopulations. Specifically, CD3^+^CD4^+^ T lymphocytes in the infected groups showed a slight increase at 12 dpi compared to levels in the control group (**Figure 7A**). Notably, CD3^+^CD8^+^ T lymphocytes showed a significant *(p < 0.05*) increase, especially in the M8-infected group, at 12 dpi, persisting slightly higher up to 28 dpi (**Figure 7B**). Additionally, CD3^+^CD4^+^CD8^+^ T lymphocytes exhibited a significant (*p < 0.05*) increase, particularly in the M8 and PJ10 infected groups; however, at low percentages compared to CD3^+^CD4^+^ and CD3^+^CD8^+^ populations (**Figure 7C**).

#### 3.4.5 Effector T lymphocytes and natural killer cells

Overall, the percentages of various T-cell responses were significantly (*p < 0.05*) induced in BAL cells at 12 dpi. Th1 (IFN-γ^+^/CD4^-^/CD8^+^) and Th17 (IL-17^+^/CD4^+^/CD8^+^) responses in the infected groups were slightly higher than those in the control groups at 12 dpi, with no significant difference observed among the infected groups (**Figures 7D, E**). Intriguingly, the frequency of cytotoxic T lymphocytes (CTL; IFN-γ^+^/CD3^+^/TCR^-^/CD8^+^) was significantly (*p < 0.05*) higher in the PJ10-infected group at 12 dpi (**Figure 7F**). To observe the alteration of NK cells in pigs upon PRRSV infection, we analyzed the frequency and pattern of two different NK-cell subsets in the BAL cell population. Interestingly, the percentages of NKp46^+^ (CD8^+^/Nkp46^+^/CD3^-^) NK cells were slightly higher in the infected group, notably significantly (*p < 0.05*) higher in the M8 infected group compared to the control group at 12 dpi (**Figure 7G**). In contrast, the percentages of NKp46^-^ (CD8^-^/Nkp46^+^/CD3^-^) NK cells in the BAL cell population of infected pigs, especially in the GGYC45 and PJ10 infected groups, were significantly (*p < 0.05*) higher at 12 dpi (**Figure 7H**).

### 3.5 Temporal profiling of T lymphocyte and natural killer cell subsets in PRRSV-2 infected pigs: insights from whole blood flow cytometric analysis

**Figure 8:**
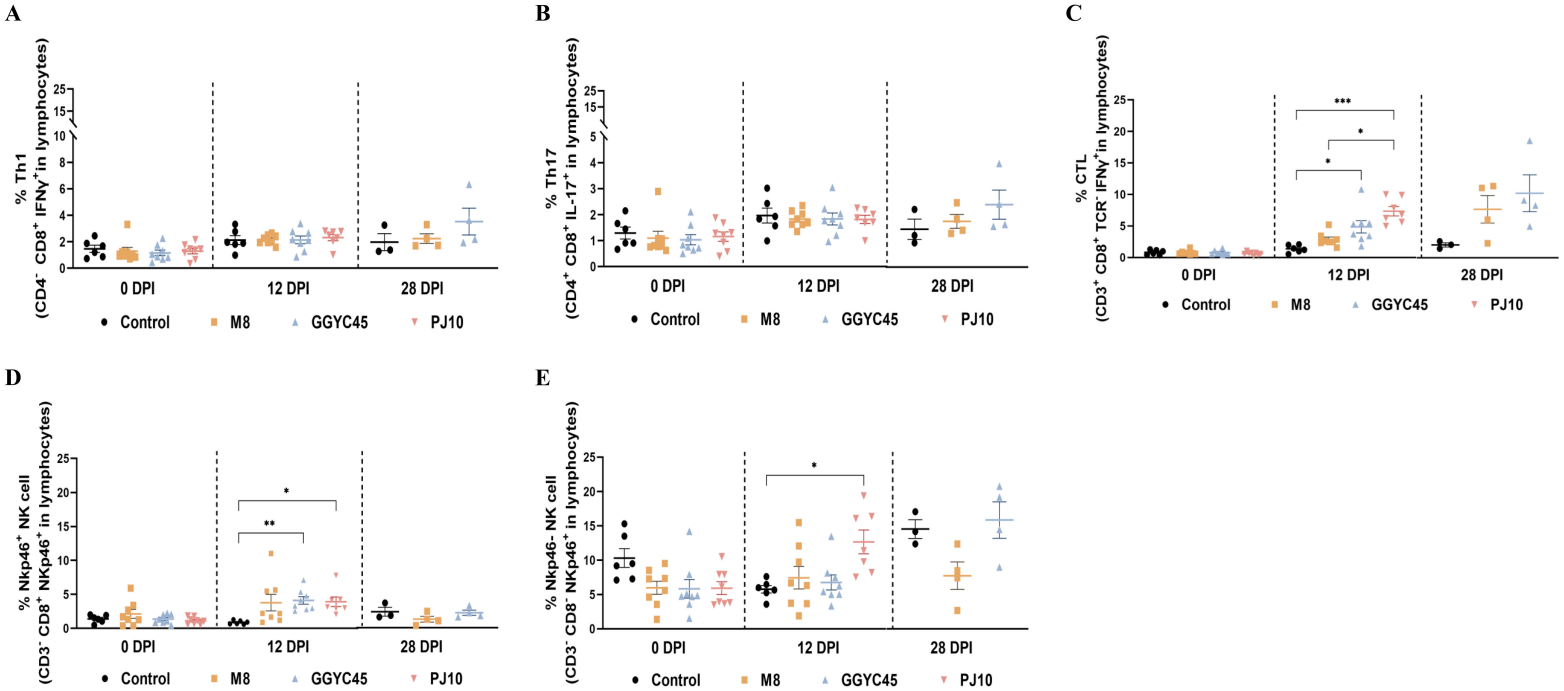
Temporal profiling of NK and T lymphocyte subsets in whole blood of PRRSV-2 infected pigs. **(A)** Thi and **(B)** Th17 responses in whole blood of PRRSV-2 infected pigs at 0, 12, and 28 days post-infection (dpi), with no significant differences observed compared to control pigs. **(C)** Cytotoxic T lymphocyte (CTL) response in pigs infected with different LKB lineage strains, including GGYC45, PJ10, and M8. A significantly increased CTL response (*p* < 0.005) was detected in GGYC45 and PJ10- infected pigs at 12 dpi, which persisted until 28 dpi, compared to the M8-infected group. **(D, E)** NK cell subset analysis in whole blood of infected pigs. A significant increase in the percentage of NKp46+ NK cells (*p* < 0.05) was observed in the GGYC45 and PJ10-infected groups at 12 dpi, whereas the NKp46- subset exhibited a higher overall magnitude of increase relative to the NKp46+ NK cells across all time points. Bars with asterisks (*) indicate significantly different values (*indicates *p* < 0.05, **indicates *p*<0.005, ***indicates *p*<0.0005).

The characteristics of systemic immunity were evaluated by analyzing NK and T-cell populations in whole blood (WB) samples collected from uninfected and infected pigs at 0, 12, and 28 dpi. The Th1 and Th17 responses in infected pigs showed no significant differences compared to those in control pigs (**Figures 8A, B**). Among the strains within the LKB lineage, pigs infected with GGYC45 and PJ10 exhibited a significantly increased CTL response (*p < 0.005*) compared to the M8-infected group at 12 dpi, which was maintained until 28 dpi (**Figure 8C**). Consistent with the results from BAL cell flow cytometry, a trend in the responses of NK cell subsets (NKp46^+^ and NKp46^-^ cells) was observed in WB from infected pigs. While the percentage of NKp46^+^ NK cell subsets significantly increased (*p < 0.05*) at 12 dpi in the GGYC45 and PJ10 infected groups, the overall magnitude of increase in NKp46^-^ NK cells was higher compared to NKp46^+^ NK cells (**Figures 8D, E**).

### 3.6 Immune checkpoint molecules in PRRSV-2 infected pigs: temporal expression and dynamics

**Figure 9:**
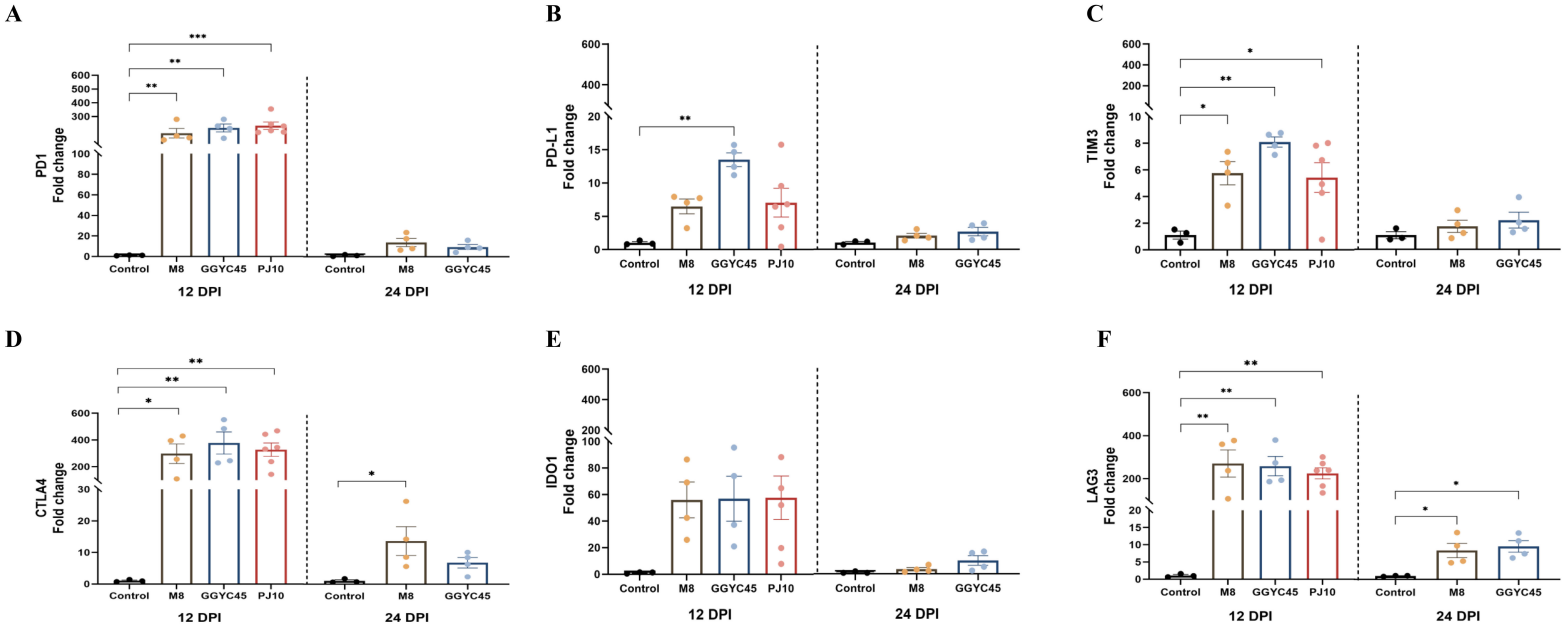
Temporal expression of immune checkpoint molecules in BAL cells of PRRSV-2 infected pigs. Expression levels of various immune checkpoint molecules in bronchoalveolar lavage (BAL) cells were analyzed in genetically diverse PRRSV-2-infected pigs. **(A)** PD1 expression levels in BAL cells from PRRSV-2 infected pigs, with a significant (*p* < 0.05) upregulation observed in PJ10 and GGYC45-infected pigs, both from lineage LKB, reaching over 200-fold compared to controls at 12 dpi. **(B)** PDL1 expression significantly increased (*p* < 0.05) in PJ10 and GGYC45-infected pigs at 12 dpi. **(C)** TIM3 expression in infected groups, showing a significant elevation (*p* < 0.05) at 12 dpi, with the GGYC45- infected group exhibiting the most pronounced increase. **(D, E)** Progressive upregulation of CTLA4 and IDO1, respectively, across infection time points, although no significant differences were observed between the infected groups. **(F)** LAG3 expression was markedly upregulated at 12 dpi, with the M8-infected group showing the highest levels compared to the other strains. Bars with asterisks (*) indicate significantly different values (*indicates *p*<0.05, **indicates *p*<0.005, ***indicates *p*<0.0005).

Overall, the expression of immune checkpoint molecules was observed to be upregulated regardless of the PRRSV strains. At 12 dpi, immune checkpoint molecules were significantly upregulated in the BAL cells of the GGYC45- and PJ10-infected groups within the LKB. Significant (*p < 0.05*) upregulation of PD1 was observed in BAL cells following PRRSV infection with levels exceeding 200-fold compared to controls at 12 dpi (**Figure 9A**). Similarly, expression of PDL1 increased at 12 dpi, with significant (*p < 0.005*) elevations observed in the GGYC45-infected group (**Figure 9B**). TIM3 expression also significantly (*p < 0.05*) increased in infected groups at 12 dpi (**Figure 9C**). A progressive upregulation of CTLA4 and IDO1 was observed. In the case of CTLA4, a fold change exceeding 250 was observed across all infected groups. IDO1 exhibited a similar trend to CTLA4; however, no statistical significance was observed (**Figures 9D, E**). Notably, LAG3 expression was significantly (*p < 0.005*) upregulated at 12 dpi in infected groups (**Figure 9F**).

## 4 Discussions

Herein, we investigated the pathogenicity and immune characteristics associated with infection with genetically distinct Korean-specific PRRSV strains. Prior to 2003, Ingelvac PRRS MLV variants (Lineage 5) predominated in the epidemiology of PRRSV-2 in Korea. Despite the use of the MLV vaccine, recombination between wild-type PRRSV and MLV variants led to the emergence of novel PRRSV-2 Korean lineages (14). In the previous study, GGYC45 was considered the potential ancestor of the current circulating LKB strain as it possessed the LKB-like ORF5 gene with other genetic compartments originating from the recombination between LKC and MLV variants (39). LKB strains, especially PJ10-like strains became one of the major PRRSV-2 populations circulating in Korea since its initial identification in 2014 (12, 14). These recombination-derived genetic differences, particularly in the ORF5 gene and non-structural regions, are hypothesized to influence viral replication, immune evasion, and tissue tropism. Such genetic alterations may partially explain the distinct pathogenic profiles observed in this study, where the PJ10 strain exhibited higher virulence compared to GGYC45 and M8, despite sharing a common LKB lineage background (23).

These genomic traits appear to translate into distinct clinical outcomes, particularly in the PJ10-infected group, as reflected in differential disease severity and virological responses Based on clinical results, the PJ10 strain belonging to LKB demonstrates more severe pathogenicity than the other Korean-type PRRSV-2 strains, evidenced by severe clinical signs, including hyperthermia decreased weight gain, and higher viral replication. Notably, moderate to severe lung consolidation accompanied by a significant decrease in ADWG, characteristic of highly pathogenic PRRSV strains, was observed in the LKB-infected group (46, 47). Moreover, a significant negative correlation (*p < 0.0001*) between ADWG and viremia AUC at 12 dpi in the PRRSV-2 infected group was observed (**Supplementary Figures 2A, B**). In general, highly virulent PRRSV strains are characterized by high temperatures and viremia at early times of infection (48). LKB strains observed high viremia accompanied by high temperature, these strains induced negatively impact the ADWG in pigs. These findings align with previous studies analyzing the association among ADWG, viremia AUC, and host immune responses such as T cell response (26, 49, 50). Additionally, PRRSV and inflammation scores of the LKB-infected groups were markedly higher than the MLV variant strain (M8). This underscores the severity of clinical symptoms in pigs infected with LKB.

PRRSV-associated brain lesions have been rarely confirmed except for PRRSV infections with highly pathogenic strains such as Chinese HP-PRRSV and PRRSV 1-4–4 Lineage 1C variants (18, 51). Notably, with virus antigens confirmed in brain tissues with IHC, pigs infected with PRRSV 1-4–4 L1C variants exhibited neurological signs including ataxia, incoordination, posterior paresis, and convulsion without any bacterial co-infection (51). Additionally, it is significant that the highly pathogenic PRRSV antigens were able to penetrate the blood-brain barrier (BBB), thereby reaching the brain (51). Intriguingly, the PRRSV-positive antigens were detected in the Virchow-Robin (perivascular) space in PJ10-infected pigs. Virchow-Robin spaces are physiological structures in normal brain parenchyma that contain interstitial fluid filled with macrophages (52). In a previous study, PRRSV-positive antigens were identified in field cases (53). The affected farm exhibited a high morbidity rate (85%) and mortality rate (12%), with clinical signs indicative of nervous system involvement, including ataxia, posterior paresis, and convulsions. Immunohistochemical analysis revealed PRRS antigens in the brain tissue of weaning pigs, with prominent brown staining of perivascular and intramural mononuclear cells in small arteries and capillaries throughout the brain. Likewise, pigs infected with the PJ10 strain exhibited neurological symptoms, including immobility, resembling those observed in PRRSV infections with highly pathogenic strains. Immunohistopathological evaluations revealed the presence of PJ10 antigens, indicating potential dissemination to the brain. Highly pathogenic PRRSV strains appear to affect multiple tissues and organs beyond the respiratory system (54, 55). Consistent with findings from previous studies, the PJ10 strain exhibits characteristics similar to those of highly pathogenic PRRSV. Therefore, detailed studies on viral infection mechanisms and host immune responses are essential to understand its pathogenesis.

To evaluate the cell population modulation within the lung in response to various PRRSV infections, collected BAL cells were analyzed using flow cytometry (FACS). Consistent with previous studies, the DC/Macrophage network in the lung environment undergoes alterations following PRRSV infection (26, 27). During the initial stages of PRRSV infection, a continuous decrease is observed in MHCII^+^ and AM cells, which are significant constituents of pulmonary alveolar macrophages (PAM) cells. In response to PRRSV infection, the host immune system initiates a defense by recruiting monocyte-derived cells to the lungs (25). The PJ10 infection resulted in significant alterations in the dendritic cell/macrophage network during the early stages of infection, including a decrease of over 50% in the monocyte population. In pigs infected with PRRSV-1 Lena, a severely disrupted monocyte network was observed from early infection stages, persisting without recovery (56). Similar findings in PJ10-infected pigs suggest that PJ10 may exhibit pathogenic characteristics comparable to those of highly pathogenic PRRSV strains, such as the Lena strain. Contrary to the marked reduction observed in AM, a noticeable increase in the influx of monocyte-derived cells was observed. Among them, a significant increase in the ratio of moM and moDC, associated with viral defense mechanisms, was observed in the PJ10-infected group. Unlike the aforementioned studies (26, 27), this study did not investigate alterations in the DC/macrophage network during the early stages of PRRSV infection (3 dpi and 7 dpi). Nevertheless, distinct patterns of monocyte collapse were observed, varying with viral strain and pathogenicity. Furthermore, the characteristics of monocyte-derived cells infiltrating the lungs differed depending on the pathogenicity of the virus. Despite the activation of a vigorous host immune response associated with monocytes following PJ10 strain infection, the PJ10-infected group exhibited severe clinical symptoms and mortality. Therefore, additional analysis was conducted on cellular immune responses, specifically focusing on T lymphocytes and NK cells.

The Cytotoxic T Lymphocyte (CTL) reaction is a host reaction to respond to virus infection (57), but interesting results were obtained in LKB-infected pigs. The PJ10 strain, which caused higher mortality in pigs, appears to have triggered both local (BAL cells) and systemic (PBMC) host immune responses to the virus; however, it is likely that these responses ultimately failed to mount an effective defense. In addition, there is a notable influx of CD3^+^CD4^+^ T cells into the lungs during the early stages of infection, less than 1% of these cells differentiate into effecter T lymphocytes (Th1, Th17). This low proportion of effector T cells suggests that an effective host immune response does not occur, which appears to be influenced by the expression of immune checkpoint molecules that inhibit T-cell activation (30, 58). In general, various immune checkpoint molecules were shown to be expressed in several cell types, and overexpression in T cells is known to cause T-cell dysfunction (59–63). Also, an analysis of immune checkpoint molecule expression in lymph nodes during PRRSV high- and low-pathogenic strain infection has already been performed (38). The characterization of immune checkpoint molecules in local organs (PAMs) has been analyzed (64). Due to PRRSV infection, the T-cell exhaustion pathway was exclusively activated. Consistent with previous findings (65), our results also demonstrated an increase in the expression of T cell exhaustion markers, including PD1, LAG3, TIM3, CTLA4, IDO1, and PDL1 which are known to downregulate T cell activation, significantly (*p < 0.05*) increased at 12 dpi. Recent studies, including single-cell transcriptomic analyses of bronchoalveolar lavage cells during PRRSV infection, have further highlighted that CTLA4 expression is significantly upregulated in alveolar macrophages, contributing to PRRSV-induced immunosuppressive environments (64, 66, 67). Integrative transcriptomic profiling also supports the critical involvement of CTLA4 in the impairment of T cell responses during PRRSV infection (65). These findings indicate that PRRSV infection involves complex mechanisms in modulating host immune responses and may represent a key factor in the failure of the host immune system to mount an effective defense against PRRSV.

Taken together, the PJ10 strain is considered a highly pathogenic variant of PRRSV-2, characterized by severe clinical symptoms, including neurological disorders, high mortality, and significant alterations in immune cell populations, particularly monocytes and T cell subsets. These observations suggest that the failure of the host immune system to effectively respond to highly pathogenic PRRSV infection is likely driven by complex immunological mechanisms. Despite the limitation of focusing on the later stages of infection, the potential role of immune checkpoint molecules in suppressing effective T-cell responses has been observed. Therefore, further research is needed to investigate the early phase of infection to fully elucidate the initial host-pathogen interactions. Moreover, the substantial upregulation of immune checkpoint molecules observed in this study highlights the need for further investigation to determine their precise role and potential as therapeutic targets for managing PRRSV-induced immunopathogenesis.

## 5 Conclusion

This study provides valuable insights into the pathogenic characteristics and immune response alterations of genetically distinct Korean PRRSV-2. Notably, the PJ10 strain which belongs to LKB exhibited severe clinical signs, including high viremia, severe lung consolidation, and significant reductions in ADWG, setting it apart from other PRRSV-2 strains. Alongside PJ10, our research also analyzed three genetically distinct PRRSV strains, which presented a challenging experimental framework and offered a comprehensive view of the varying pathogenic profiles. Despite belonging to the same LKB lineage, the patterns of DC/macrophage network alterations differed between the GGYC45 and PJ10 strains, with PJ10 showing more severe disruptions. This finding aligns with clinical signs, including ADWG, high temperature, and highest viral load, which were more severe in PJ10-infected pigs. Although the host immune response, such as an increase in effector T cells like cytotoxic T lymphocytes (CTLs) in BAL cells (BALc) and Whole blood (WB), was upregulated, it failed to prevent mortality associated with PJ10 infection. Furthermore, the upregulation of immune checkpoint molecules suggests a critical role in the suppression of effective T-cell responses, which is considered to be the reason for the inadequate defense against PRRSV. These findings highlight the importance of further investigation into the immunopathogenesis of PRRSV, particularly the mechanisms of infection and host immune evasion, better to understand the underlying reasons for inadequate immune responses.

## Data Availability

The datasets presented in this study can be found in online repositories. The names of the repository/repositories and accession number(s) can be found in the article/**Supplementary Material**.
