# Supplementary material for: Immunopathological features of highly pathogenic Korean Lineage B PRRSV-2: insights into virulence indicators and host immune responses
Source: Front Immunol. 2025 Jun 18;16:1599468. doi: 10.3389/fimmu.2025.1599468 (PMC12213469; doi:10.3389/fimmu.2025.1599468)
Supplement: Supplementary file 1 [file Image1.pdf]

Supplementary figure 1

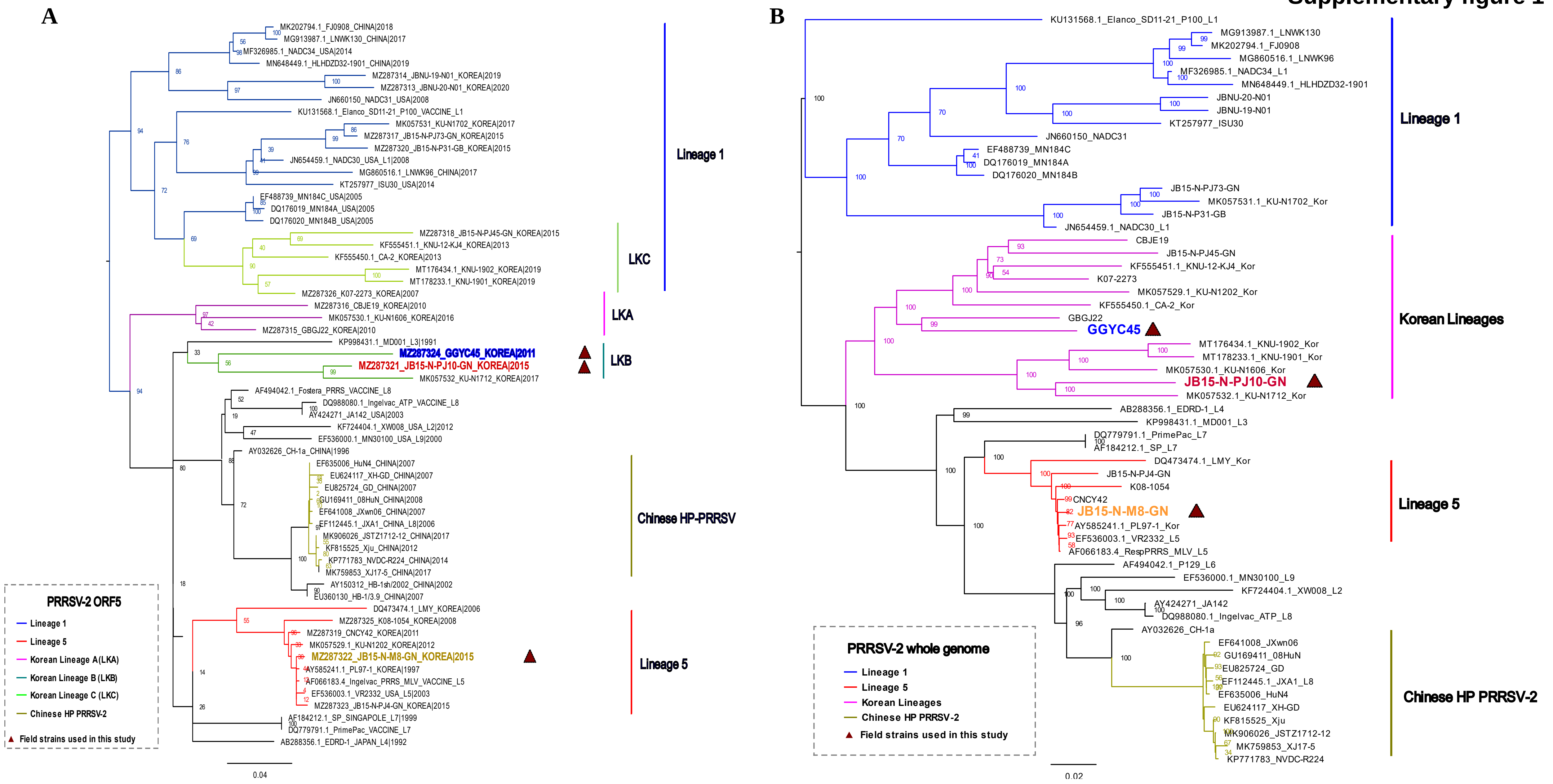

Supplementary Figure 1: Phylogenetic analysis of Korean Lineage B (LKB) PRRSV-2

Phylogenetic tree of (A) ORF5 and (B) whole-genome sequences of Korean Lineage B (LKB) strains. LKB and reference strains used in this study are indicated in bold and color-coded; M8 (Brown), GGYC45 (Blue), and PJ10 (Red). Trees were generated by RAxML-NG with 1,000 bootstrap replicates using the GTRGAMMA nucleotide substitution model
