# Supplementary material for: Immunopathological features of highly pathogenic Korean Lineage B PRRSV-2: insights into virulence indicators and host immune responses
Source: Front Immunol. 2025 Jun 18;16:1599468. doi: 10.3389/fimmu.2025.1599468 (PMC12213469; doi:10.3389/fimmu.2025.1599468)
Supplement: Supplementary file 2 [file Image2.pdf]

Supplementary figure 2

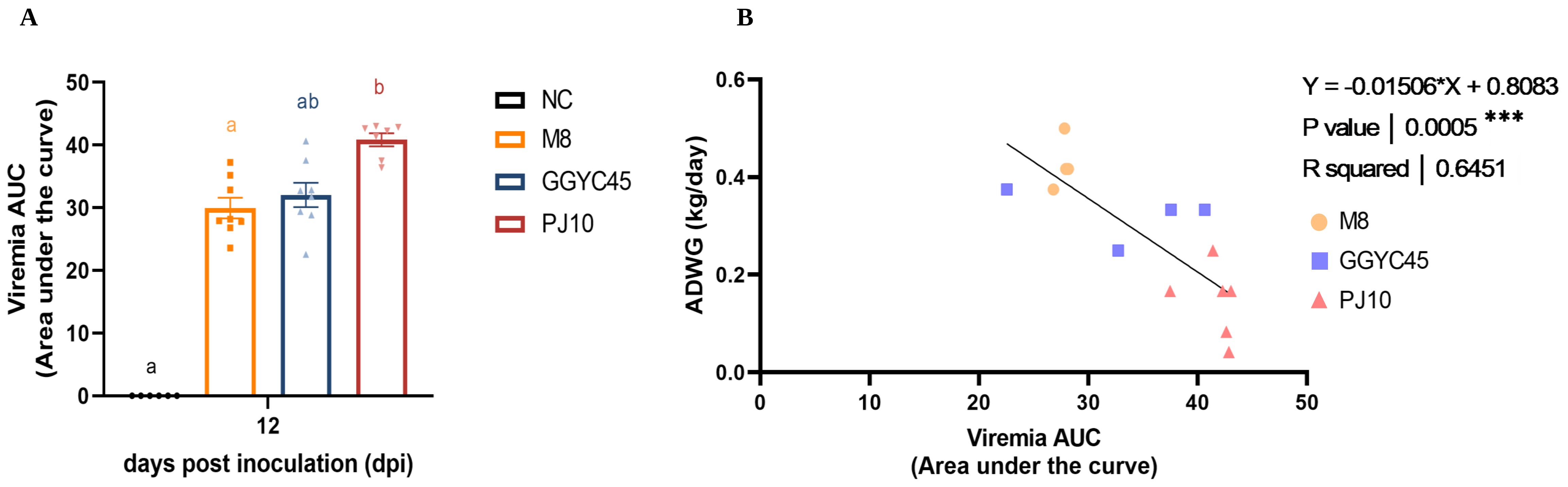

Supplementary Figure 2: Correlation between viremia AUC and average daily weight gain (ADWG) in PRRSV-2 infected pigs
(A) Pigs infected with the PRRSV-2 PJ10 strain exhibited a significantly higher viremia Area Under the Curve (AUC) compared to other strains. (B) A significant negative correlation (*p < 0.0001*) was observed between ADWG and viremia AUC at 12 days post-infection (dpi) across the PRRSV-2 infected groups, indicating that higher viremia was associated with a greater reduction in weight gain. Different lowercase letters (a, b, c) above the bars indicate statistically significant differences between groups at the same time point ($p < 0.05$), as determined by ANOVA followed by post-hoc analysis.
